# Supplementation with porcine placenta extract reduces negative emotions and enhances positive emotions in healthy adults: a randomized, double-blind, placebo-controlled study

**DOI:** 10.3389/fnut.2025.1570736

**Published:** 2025-07-23

**Authors:** Naoya Morita, Eiichi Hirano

**Affiliations:** ^1^Department of Marketing, Japan Bio Products, Co., Ltd., Tokyo, Japan; ^2^Department of Medical Affairs, Japan Bio Products, Co., Ltd., Tokyo, Japan

**Keywords:** mental health, placental extract, supplementation, Profile of Mood States, 2nd Edition (POMS-2), intervention study, double-blind, randomized

## Abstract

**Background:**

Mental health conditions such as depression, anxiety, and stress represent significant global health challenges. According to the World Health Organization, there is an urgent need for safe and effective interventions to alleviate this burden. However, existing approaches to address these conditions remain insufficient. Placenta extract, obtained by enzymatic digestion of the placenta, has demonstrated efficacy in improving menopausal symptoms and may represent a novel interventional option.

**Objective:**

This study aimed to evaluate the effects of porcine or equine placenta extract (pPE or ePE, respectively) intake on stress levels in healthy adults.

**Methods:**

This was a 12-week, randomized, double-blind, placebo-controlled study (ClinicalTrials Identifier: UMIN000053020; https://center6.umin.ac.jp/cgi-open-bin/ctr_e/ctr_view.cgi?recptno=R000060483). This study was conducted among Japanese men and women aged 20–65 years with total mood disturbance (TMD) scores of 40–75, as assessed by the Profile of Mood States, 2nd Edition. The primary endpoint was TMD score, while secondary endpoints included fatigue, quality of life, sleep quality, autonomic function, and stress hormone levels.

**Results:**

The pPE group showed significant reductions in TMD scores and improvements in Anger-Hostility, Confusion-Bewilderment, Vigor-Activity, and Friendliness subscales compared to the placebo group. In contrast, no significant improvement in TMD was observed in the ePE group compared to the placebo group. No significant differences in the secondary endpoints were observed among any of the groups.

**Conclusion:**

These findings suggest that placenta extract intake may reduce negative emotions while promoting positive ones.

**Clinical review registration:**

https://center6.umin.ac.jp/cgi-open-bin/ctr_e/ctr_view.cgi?recptno=R000060483, identifier UMIN000053020.

## 1 Introduction

In recent years, shifts in social structure have contributed to increased psychological stress and a growing prevalence of mental health issues such as mood disorders and anxiety (1). According to the World Health Organization, mental health conditions such as depression, anxiety, stress, and burnout constitute a significant portion of the “global burden of disease” and are recognized as a “health epidemic of the 21st century.” This emphasizes the urgent need for safe and effective interventions to alleviate such challenges (2–4). The World Health Organization initially addressed these concerns through the “Mental Health Action Plan 2013–2020,” which was updated in 2021 and extended to 2030 to further its objective (5).

A systematic review and meta-analysis estimated the global prevalence of depression at 28.0%, anxiety at 26.9%, post-traumatic stress symptoms at 24.1%, stress at 36.5%, psychological distress at 50.0%, and sleep disturbances at 27.6% (6). However, it should be noted that these figures reflect the specific social context of the Coronavirus Disease 2019 pandemic. Although pharmacotherapy, such as antidepressants, is available for these conditions, its efficacy is often limited in patients with mild or moderate symptoms (7). Moreover, pregnant women are frequently reluctant to use psychotropic medications due to concerns about potential risks (8). Therefore, it is important to incorporate preventive measures into daily life to reduce stress and address mild psychiatric issues. Nutritional interventions, in particular, are safe and accessible options. Recent studies have demonstrated the efficacy of various foods and supplements in mental health, reducing stress, and enhancing mood. For instance, gamma-aminobutyric acid, theanine, and rosemary extract have been reported to improve mental health, alleviate mental stress, reduce negative mood states, and boost motivation (9–11). A blend of five amino acids [serine, alanine, glutamic acid, aspartic acid, and tyrosine (Tyr)] has been reported to significantly improve motivation and cognitive function (12). Collagen peptide supplementation has been associated with improved mood states such as reduced fatigue and enhanced vitality (13). Daily consumption of chicken essence for 1–4 weeks (W) may relieve mental fatigue in healthy individuals (14). In addition, a systematic review and meta-analysis suggests that probiotic supplementation provides statistically significant improvements in psychological symptoms (15).

Placenta extract (PE), derived through enzymatic digestion and acid hydrolysis of the placenta, is rich in peptides, amino acids, and nucleobases. PE can significantly improve anxiety as well as symptoms such as “irritability,” “depression,” and “fatigue,” as measured by the Simplified Menopausal Index score (16, 17). In animal models of physical fatigue, PE has been reported to alleviate fatigue and reduce biochemical parameters such as lactate, lactate dehydrogenase, glucose, creatine kinase, blood urea nitrogen, cortisol and inflammatory cytokines (18). In healthy adults (84 healthy men and women aged 30–60 years), PE has demonstrated significant improvements in fatigue-related parameters and subjective symptoms (19). Although the active components in PE responsible for these effects have not been fully identified, glycylleucine (Gly-Leu) and leucylglycine (Leu-Gly)—dipeptides that promote brain-derived neurotrophic factor secretion—are likely candidates (20). Moreover, a recent study reported that a 4-week course of PE improved psychological distress in middle-aged and older women (21). These findings suggest that PE may enhance vitality and reduce fatigue, particularly in individuals experiencing low energy levels and increased fatigue.

Phenylalanine, Tyr, tryptophan, and kynurenine, the precursors of neurotransmitters and their metabolites, are associated with mental stress. Acute mental stress has been reported to increase kynurenine/tryptophan in healthy college students (22). Maintaining neurological function is critical for mental health, and an association between neuroaxonal damage and fatigue has been observed in multiple sclerosis (23). These findings suggest that amino acids influence neuropsychiatric conditions such as fatigue and mental disorders. Amino acid supplements have demonstrated efficacy in reducing symptoms of depression and other mental health issues, as the amino acids absorbed by the body are converted into neurotransmitters that alleviate such conditions (24). A systematic review concluded that Tyr intake may have beneficial effects on psychological function in healthy adults (25). Furthermore, a 4-week intervention with a combination of five amino acids (serine, alanine, glutamic acid, aspartic acid, and Tyr) was reported to significantly improve motivation and cognitive function during the recovery period after a stressful mental task (12).

Based on the aforementioned findings, PE is hypothesized to have a potential beneficial role in the regulation of mental health. This clinical trial was conducted to evaluate the effects of porcine and equine placental extracts on mood states, including both negative and positive affect, as the primary outcome in healthy men and women.

## 2 Materials and methods

### 2.1 Study design and participants

This randomized, double-blind, placebo-controlled, parallel-group study was conducted at the Seishinkai Takara Clinic (Tokyo, Japan) from November 2023 to July 2024. The study was managed by the contract research organization ORTHOMEDICO Inc. (Tokyo, Japan). A similar study examining the effects of placental extract supplements was used as a reference, with the initial target set at 90 participants (17). To account for potential dropouts during the study period, an additional 12 participants were enrolled, bringing the total number of participants to 102. Participants were screened for eligibility based on the inclusion and exclusion criteria through interviews conducted by a supervising physician prior to the enrollment. Eligible participants were randomly assigned to one of three groups—placebo, pPE, or ePE—groups in a 1:1:1 ratio using stratified randomization generated by a computer. Randomization was stratified based on Total Mood Disturbance (TMD) scores, sex, and age after loading. An independent allocation manager, unaffiliated with participant eligibility determination, data collection, or data analysis, handled the randomization process and maintained confidentiality of the allocation list until the study’s database was unlocked. The manufacturer of the test supplements labeled identification numbers on the test supplements before providing them to the contract clinical trial organization. To ensure blinding, the placebo and PE products were indistinguishable in appearance, and the participants, investigators, and research staff remained unaware of group assignments throughout the study. Participants were enrolled based on the following inclusion criteria: Japanese individuals of both sexes, aged 20–65 years, with Profile of Mood States 2nd Edition (POMS2) scores ranging from 40 to 75 points. The exclusion criteria were as follows: a current or past history of malignancy, heart failure, myocardial infarction, pacemaker or implantable cardioverter-defibrillator use, arrhythmia, liver disease, chronic kidney disease, cerebrovascular disease, rheumatic disease, diabetes, dyslipidaemia, or hypertension. Participants with allergies to any medications or test foods, especially those involving horsemeat or pork, and dietary supplements, including herbal supplements were also excluded. Individuals with a history of psychiatric disorders, such as depression or attention deficit hyperactivity disorder, or those with irregular sleeping patterns (e.g., night shifts), were not eligible. Additionally, participants with irregular lifestyle habits related to eating, exercise, or sleep, those undergoing treatment for chronic fatigue syndrome or menopause, and those engaged in heavy weightlifting or other physical labor were excluded. Additionally, individuals with premenstrual syndrome or premenstrual dysphoric disorder were excluded. Pregnant or lactating women, as well as those planning to become pregnant during the study period, were not enrolled. The use of dietary supplements known to affect fatigue and stress (S-allylcysteine, gamma-aminobutyric acid, lactic acid bacteria, reduced coenzyme Q10 from *Euglena gracilis Paramylon*, docosahexaenoic acid, eicosapentaenoic acid, astaxanthin, citric acid, L-theanine, black soybean polyphenols, 5-aminolevulinic acid phosphate, and anserine) was a ground for exclusion. Participants undergoing counseling or psychotherapy, those currently or previously treated with hormone therapy, and those who had participated in another clinical trial within 28 days prior to providing consent were also excluded. Finally, individuals deemed unsuitable for the study by the investigator were not included. The compliance of study participants was monitored by means of interviews, diaries and dietary surveys.

### 2.2 Intervention and study products

Three different capsule powders were prepared as test supplements: porcine placenta extract (pPE; 200 mg), equine placenta extract (ePE; 200 mg), and a placebo formulation (200 mg). These intervention products are a placenta extract obtained by enzymatic hydrolysis of each placentas. The main components of these extracts are amino acids and peptides. The placebo consisted of starch, cornstarch, stearic acid, calcium, and micronized silica. All three test supplements were indistinguishable in appearance, size, shape, color, odor, and taste to ensure blinding. Participants consumed five capsules daily (equivalent to 1,000 mg) of either placebo or PE product with lukewarm water after breakfast for 12 weeks (12 W). They were also instructed to continue their regular lifestyle during the study. Adherence to supplementation regimen was monitored through diet control charts, where participants recorded any leftover capsules.

### 2.3 Study procedures and data collection

Mood states were assessed using the Japanese version of the POMS2, which evaluates TMD, and includes subscales for Tension-Anxiety, Depression-Dejection, Anger-Hostility, Vigor-Activity, Fatigue-Inertia, Confusion-Bewilderment, and Friendliness (26, 27). Fatigue was assessed using the fatigue Visual Analog Scale (VAS) recommended by Japanese Society for Fatigue Science (28). Briefly, participants marked a position on a 100-mm straight line, where the left end (0) represented the least fatigue and the right end represented the worst fatigue (complete exhaustion preventing any activity). The distance (mm) from the left end to the marked position was evaluated as a measure of fatigue severity. Health-related quality of life was assessed using the Medical Outcome Study (MOS) Short-Form 36-Item Health Survey (SF36v2), which provides summary scores for physical (Physical Component Summary; PCS) and mental (Mental Component Summary; MCS) aspects of quality of life, along with subscales for Role-Social Component Summary, Physical functioning, Role Physical, Bodily Pain, General Health, Vitality, Social Functioning, Role Emotional, and Mental Health. Summary scores and subscales were calculated based on national norms (29, 30). Sleep quality was assessed using the Oguri-Shirakawa-Azumi Sleep Inventory, Middle-age and Aged version (OSA-MA) (31). Participants completed the inventory on the 3 days immediately preceding and including the test day (the day of the test, the day before the test, and the day before the day before the day of the test). The questionnaire asked about “sleepiness on rising,” “initiation and maintenance of sleep,” “frequent dreaming,” “refreshing,” and “sleep length,” with measurements averaged over the 3 days to calculate Zc values. Autonomic function was assessed using the Vital monitor (VM-302), which measured low frequency, high frequency, low frequency/high frequency ratio, average heart rate, maximum heart rate, minimum heart rate, total power (TP), coefficient of component variance total power (ccvTP), autonomic function deviation, and functional age (32). To ensure participant safety, blood assessments for liver and kidney function were conducted both before and after the intervention. Additionally, changes in blood pressure, body mass index (BMI), and self-reported adverse events were monitored throughout the study duration.

### 2.4 Study endpoints

The primary outcome of the study was the change in TMD scores as assessed by POMS2 after 12 W of consumption. The secondary outcomes of the study included: Change in TMD scores as assessed by POMS2 after 12 W of intake in the ePE group. Changes in Tension-Anxiety, Depression-Dejection, Anger-Hostility, Vigor-Activity, Fatigue-Inertia, Confusion-Bewilderment, and Friendliness as assessed using POMS2 after 12 W of intake. Changes in fatigue levels as assessed by VAS after 12 W of intake. Changes in quality of life: PCS, MCS, RCS, Physical functioning, Role Physical, Bodily Pain, General Health, Vitality, Social Functioning, Role Emotional, and Mental Health as assessed using SF-36 after 12 W of intake. Changes in waking sleepiness, falling and staying asleep, dreaming, fatigue recovery, and sleep duration as assessed using the OSA-MA after 12 W of intake. Changes in plasma cortisol and dehydroepiandrosterone sulfate (DHEA-S) after 12 W of intake. Changes in Autonomic measures of low frequency, high frequency, low frequency/high frequency, mean heart rate, maximum heart rate, minimum heart rate, TP, ccvTP, autonomic function deviation after 12 W of intake.

### 2.5 Statistical analysis

Results were presented as mean ± standard deviation (SD). Statistical analyses were performed by statisticians from the contract research organization using IBM SPSS Statistical software version 23 (IBM Japan, Ltd., Tokyo, Japan). For each participant analyzed, demographic characteristics were recorded and disaggregated. A chi-squared test was used to compare groups based on sexual distribution. Analysis of covariance (ANCOVA) was used to compare age, BMI, systolic and diastolic blood pressure, the Beck Depression Inventory-2 (BDI-2) total score, and TMD scores between the groups. Efficacy and safety analyses were performed using the Full Analysis Set under an intention-to-treat basis. ANCOVA was used for post-intervention comparisons between groups, treating pre-trial values as covariates and group allocation as a factor. All statistical tests were two-tailed, and a *p* < 0.05 was considered indicative of statistical significance. Due to budgetary constraints at the time the study was planned, it was not possible to perform a formal *a priori* sample size calculation. We acknowledge this as a limitation. We conducted a *post hoc* power analysis based on the observed effect size, which gave a result of 53%. To evaluate individual-level changes in mood, we calculated the change in total POMS2 score from baseline to post-intervention. Participants were categorized as having experienced a beneficial change, no change, or a harmful change, based on a threshold of ± 0.5 standard deviations of the baseline score, consistent with commonly accepted definitions of minimal clinically important difference (MCID).

## 3 Results

### 3.1 Study population

**FIGURE 1:**
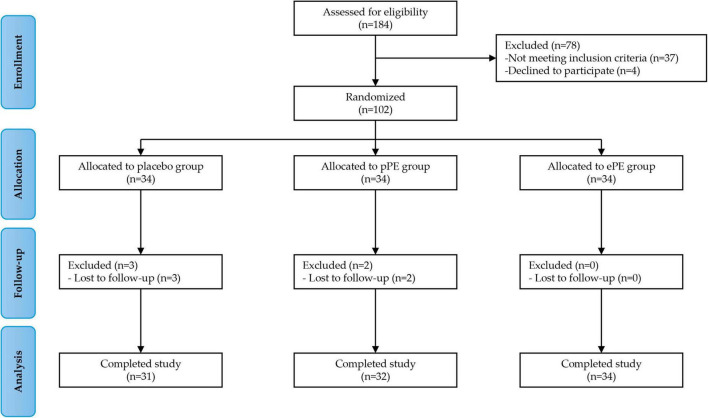
Participant enrolment and study design.

A total of 102 eligible participants were enrolled in Phase 1 of the study. Participants were randomly assigned in equal numbers to one of the three groups: the placebo group (34 participants), the pPE group (34 participants), and the ePE group (34 participants). All assigned participants received the intervention. All participants began the intervention, but five participants did not complete the study. Specifically, three participants in the placebo group and two in the pPE group did not complete the intervention because they did not return to the clinic for the 12-week follow-up visit (see the full experimental plan in Figure 1). Baseline characteristics and questionnaire scores at baseline are shown in Table 1. The mean age of participants in the placebo, pPE and ePE groups was 46.0 ± 11.5, 45.4 ± 11.9 and 44.6 ± 11.3, respectively. The number of male and female participants in the placebo, pPE and ePE groups was 10 and 21, 9 and 23, and 10 and 24, respectively (Table 1). The balanced baseline characteristics across the groups ensured comparability and minimized bias in the experimental results.

**TABLE 1:** Baseline characteristics of the study participants.

Variable	Placebo group (*n* = 31)	pPE group (*n* = 32)	ePE group (*n* = 34)
Number of subjects (male/female)	31 (10/21)	32 (9/23)	34 (10/24)
Age (years)	46.0 ± 11.5	45.4 ± 11.9	44.6 ± 11.3
BMI (kg/m^2^)	26.1 ± 3.4	22.5 ± 4.4	22.5 ± 5.6
Systolic BP (mmHg)	116.0 ± 16.0	117.3 ± 14.5	118.6 ± 20.1
Diastolic BP (mmHg)	76.1 ± 12.6	75.8 ± 10.6	77.6 ± 13.0
Total BDI2 score	15.5 ± 7.9	14.2 ± 5.9	14.4 ± 6.5
TMD	53.7 ± 7.9	52.4 ± 6.4	53.8 ± 7.4

The number of participants is presented as n (male/female), and comparisons between groups were performed using the χ^2^ test. Other data are expressed as mean ± SD, and comparisons between groups were conducted using ANCOVA. BDI2, Beck Depression Inventory; BMI, body mass index; BP, blood pressure; TMD, Total Mood Disturbance; SD, standard deviation.

### 3.2 Primary outcome: changes in Profile of Mood States, 2nd Edition (POMS2) scores and subgroup analysis

TMD is calculated from a total of seven scales, five of which indicate negative mood states: Anger-hostility, confusion-bewilderment, depression-dejection, fatigue-inertia and tension-anxiety. Two additional scales indicate positive mood states: Vigor-Activity and Friendliness. The TMD score, the primary validation criterion, decreased after 12 weeks in all groups: placebo, pPE, and ePE (Table 2). In addition, the 12 W TMD scores of the pPE group showed a significantly greater reduction compared to the placebo group (*p* = 0.044), whereas the decrease in the ePE group compared to the placebo group was not statistically significant (Table 2). Negative mood state scores, including Anger-Hostility, Confusion-Bewilderment, Depression-Dejection, Fatigue-Inertia, and Tension-Anxiety, decreased at 12 W across all groups. In addition, a significant difference was observed in the pPE group compared to the placebo group in Anger-Hostility and Confusion-Bewilderment at 12 W (Anger-Hostility: *p* = 0.049, Confusion-Bewilderment: *p* = 0.0011) (Table 2). However, there was no significant difference between the placebo group and the ePE group at 12 W. Scores on the positive mood state scale, Vigor-Activity increased in all groups at 12 W (Table 2). Another positive mood state scale, Friendliness showed no change in the placebo group but increased in both the pPE and ePE groups (Table 2). A significant difference was observed between the pPE group and the placebo group in terms of Vigor-Activity and Friendliness at 12 weeks (*p* = 0.025 and *p* = 0.020, respectively), but no such difference was observed between the ePE group and the placebo group (Table 2). Moreover, Cronbach’s alpha values calculated in our study sample for POMS2, SF-36, and OSA-MA questionnaires at baseline (0 W) were almost superior to 0.8, indicating a good internal consistency and supporting the reliability of the measures in our sample.

**TABLE 2:** Changes in Profile of Mood States, 2nd Edition (POMS2) scores among participants.

Variable		Placebo group	pPE group					ePE group					Cronbach’s alpha
		Mean ± SD	Mean ± SD	MD (vs. placebo group)	SE (vs. placebo group)	95% CI (vs. placebo group)	*p*-value (vs. placebo group)	Mean ± SD	MD (vs. placebo group)	SE (vs. placebo group)	95% CI (vs. placebo group)	*p*-value (vs. placebo group)	
TMD	0 W	53.7 ± 7.9	52.4 ± 6.4	−1.3	1.8	−4.9 ∼ 2.3	0.462	53.8 ± 7.4	0.1	1.9	−3.7 ∼ 3.9	0.965	0.932
	12 W	51.5 ± 10.4	47.3 ± 7.5	−2.8	1.4	−5.6 ∼−0.1	0.044*	49.1 ± 10.2	−2.5	1.8	−6.1 ∼ 1.2	0.180	0.956
Anger-hostility	0 W	50.9 ± 10.1	48.9 ± 7.0	−1.9	2.2	−6.3 ∼ 2.5	0.383	50.8 ± 10.6	−0.1	2.6	−5.2 ∼ 5.0	0.967	0.892
	12 W	50.4 ± 11.0	45.6 ± 6.2	−3.6	1.8	−7.1 ∼ 0.0	0.049*	47.4 ± 9.8	−2.9	2.0	−6.9 ∼ 1.0	0.142	0.917
Confusion-bewilderment	0 W	54.7 ± 8.7	53.6 ± 7.6	−1.1	2.1	−5.3 ∼ 3.0	0.580	54.6 ± 7.8	−0.1	2.1	−4.2 ∼ 4.0	0.965	0.713
	12 W	53.9 ± 11.3	48.4 ± 8.4	−4.4	1.7	−7.8 ∼−1.0	0.011*	51.2 ± 10.8	−2.6	1.8	−6.3 ∼ 1.1	0.167	0.874
Depression-dejection	0 W	53.2 ± 9.0	50.9 ± 6.9	−2.3	2.0	−6.4 ∼−1.8	0.265	52.2 ± 7.7	−1.0	2.1	−5.2 ∼ 3.2	0.638	0.893
	12 W	50.9 ± 9.8	48.1 ± 6.8	−1.0	1.4	−3.7 ∼−1.1	0.474	49.4 ± 9.5	−0.8	1.7	−4.2 ∼ 2.7	0.666	0.934
Fatigue-inertia	0 W	51.8 ± 9.5	53.7 ± 8.1	1.9	2.2	−2.6 ∼−6.4	0.395	53.3 ± 10.0	1.5	2.4	−3.4 ∼ 6.3	0.541	0.853
	12 W	50.8 ± 9.1	49.6 ± 8.0	−2.4	1.6	−5.7 ∼−0.9	0.147	50.4 ± 9.8	−1.2	1.9	−5.0 ∼ 2.6	0.524	0.876
Tension-anxiety	0 W	54.4 ± 10.1	52.0 ± 8.6	−2.4	2.4	−7.1 ∼−2.4	0.318	53.7 ± 8.6	−0.7	2.3	−5.4 ∼ 4.0	0.772	0.853
	12 W	51.9 ± 10.3	49.5 ± 8.9	−0.6	1.7	−4.0 ∼−2.8	0.713	48.9 ± 10.3	−2.5	2.0	−6.5 ∼ 1.4	0.201	0.890
Vigor-activity	0 W	47.3 ± 9.5	45.7 ± 7.8	−1.5	2.2	−4.0 ∼−2.8	0.485	44.9 ± 8.1	−2.4	2.2	−6.8 ∼ 2.0	0.285	0.908
	12 W	50.3 ± 9.9	53.7 ± 10.0	4.5	2.0	0.6 ∼−8.4	0.025*	50.7 ± 9.9	1.9	2.0	−2.1 ∼ 6.0	0.346	0.920
Friendliness	0 W	51.4 ± 10.4	50.1 ± 9.6	−1.3	2.5	−6.3 ∼−3.8	0.618	47.3 ± 8.7	−4.1	2.4	−8.9 ∼ 0.6	0.089	0.783
	12 W	51.3 ± 10.0	54.7 ± 8.5	4.2	1.7	0.7 ∼−7.7	0.020*	49.9 ± 10.6	0.9	2.3	−3.6 ∼ 5.5	0.676	0.816

Data are given as mean ± standard deviation (SD). Statistical analysis was performed using ANCOVA for between-group comparisons after 12 weeks. pPE, porcine placenta extract; MD, Mean difference between groups. SE; Standard error; CI, Confidential interval; 0 W, baseline; 12 W, 12 weeks; TMD, Total Mood Disturbance.

*p < 0.05 vs. placebo group.

For TMD, Anger-Hostility, Confusion-Bewilderment, Vigor-Activity, and Friendliness, which were positive results on the primary endpoint, we conducted a subgroup analysis classified by participant sex. TMD scores by sex decreased at week 12 for the female placebo group, female pPE group, male placebo group, and male pPE group (Supplementary Table S1). Although the TMD scores of the pPE group decreased at week 12 compared to the placebo group for both sexes, the difference was not statistically significant (Supplementary Table S1). Anger-Hostility scores for each sex decreased at week 12 for the female placebo group, female pPE group, male placebo group, and male pPE group (Supplementary Table S1). Anger-Hostility scores in the pPE group decreased at week 12 for both sexes compared to the placebo group, but the difference was not statistically significant (Supplementary Table S1). Although Confusion-Bewilderment scores by sex increased at week 12 in the female placebo group, they significantly decreased at week 12 in the female pPE group, the male placebo group, and the male pPE group (Supplementary Table S1). Confusion-Bewilderment scores in the pPE group of both sexes were reduced at week 12 compared to the placebo group, with a statistically significant difference in the female group (*p* = 0.008) (Supplementary Table S1). Vigor-Activity scores by sex increased at week 12 for the female placebo group, female pPE group, male placebo group, and male pPE group (Supplementary Table S1). Vigor-Activity scores increased for each sex in the pPE group at week 12 compared to the placebo group, with a significant increase in the male pPE group (*p* = 0.006) (Supplementary Table S1). Friendliness-scores by sex decreased at week 12 in the female placebo group but increased at week 12 in the female pPE group, the male placebo group, and the male pPE group. Furthermore, the Friendliness-score increased in the pPE group for both sexes compared to the placebo group, with a statistically significant increase observed in females (*p* = 0.041) (Supplementary Table S1).

Furthermore, we analyzed these responses on an individual basis. In the placebo group, 45% of participants showed a beneficial change in TMD POMS2 scores, 39% showed no meaningful change and 16% showed an adverse change. In contrast, 69% of the pPE group showed a beneficial change, 25% showed no meaningful change and 6% showed an adverse change (Supplementary Table S2). For the other measures, the proportion of beneficial changes was 16–39% in the placebo group and 22–56% in the pPE group. The proportion of participants with no meaningful changes was 45–71% in the placebo group and 34–72% in the pPE group. The proportion of adverse changes was 13–23% in the placebo group and 1–2% in the pPE group (Supplementary Table S2).

### 3.3 Secondary outcomes and safety assessment

#### 3.3.1 Changes in the fatigue as assessed by visual analog scale (VAS) among participants

The VAS, the secondary validation criterion, decreased at 12 W in all groups: placebo, pPE, and ePE with reductions observed within each group (Table 3). No significant differences were noted between the placebo group and either the pPE or ePE group at 12 W (Table 3).

**TABLE 3:** Changes in fatigue, as assessed by the visual analog scale (VAS), among participants.

	Placebo group	pPE group					ePE group				
	Mean ± SD	Mean ± SD	MD (vs. placebo group)	SE (vs. placebo group)	95% CI (vs. placebo group)	*p*-value (vs. placebo group)	Mean ± SD	MD (vs. placebo group)	SE (vs. placebo group)	95% CI (vs. placebo group)	*p*-value (vs. placebo group)
0 W	52.5 ± 22.8	58.3 ± 17.1	5.7	5.1	−4.5 ∼ 15.9	0.268	52.6 ± 20.8	0.0	5.4	−10.9 ∼ 10.9	0.998
12 W	37.1 ± 23.4	39.4 ± 23.0	0.2	5.7	−11.1 ∼ 11.6	0.967	40.7 ± 24.7	3.5	5.8	−8.0 ∼ 15.1	0.542

Data are given as mean ± standard deviation (SD). Statistical analysis was performed using ANCOVA for between-group comparisons after 12 weeks. pPE, porcine placenta extract; MD, Mean difference between groups; SE, Standard error; CI, Confidential interval; 0 W, baseline; 12 W, 12 weeks.

#### 3.3.2 Changes in the quality of life using SF-36 among participants

Health-related quality of life (QOL) scores were assessed using the SF-36, which included eight subscales. No changes in physical functioning were observed in the placebo group, but improvements were seen in the group that ingested placenta (Table 4). For physical functioning, a significant increase was observed only in the ePE group (Table 4). Increases in scores were observed in the other seven items: role physical, bodily pain, general health, vitality, social functioning, role emotional, and mental health. No statistically significant differences were observed between the placebo group and either the pPE or ePE group at 12 W (Table 4).

**TABLE 4:** Changes in quality of life, as assessed using the SF-36, among participants.

Variable		Placebo group	pPE group					ePE group				
		Mean ± SD	Mean ± SD	MD (vs. placebo group)	SE (vs. placebo group)	95% CI (vs. placebo group)	*p*-value (vs. placebo group)	Mean ± SD	MD (vs. placebo group)	SE (vs. placebo group)	95% CI (vs. placebo group)	*p*-value (vs. placebo group)
Physical functioning	0 W	51.9 ± 6.7	51.8 ± 4.6	−0.2	1.5	−3.1 ∼ 2.7	0.906	50.6 ± 6.5	−1.3	1.6	−4.6 ∼ 2.0	0.430
	12 W	51.8 ± 6.8	53.0 ± 5.4	1.4	1.2	−0.9 ∼ 3.7	0.237	53.3 ± 4.6	2.2	1.2	−0.2 ∼ 4.5	0.072
Role physical	0 W	44.0 ± 10.4	46.3 ± 7.9	2.4	2.3	−2.3 ∼ 7.1	0.310	45.9 ± 10.1	2.0	2.5	−3.1 ∼ 7.1	0.441
	12 W	48.4 ± 8.7	49.5 ± 7.2	0.6	2.0	−3.4 ∼ 4.5	0.780	50.2 ± 6.9	1.1	1.8	−2.4 ∼ 4.6	0.536
Bodily pain	0 W	46.1 ± 9.6	47.9 ± 10.1	1.8	2.5	−3.1 ∼ 6.8	0.467	46.5 ± 9.4	0.4	2.2	−4.3 ∼ 5.2	0.856
	12 W	49.4 ± 8.8	51.7 ± 8.9	1.7	2.1	−2.5 ∼ 6.0	0.409	50.0 ± 9.8	0.5	2.2	−3.9 ∼ 4.9	0.824
General health	0 W	51.6 ± 6.6	50.5 ± 6.8	−1.1	1.7	−4.5 ∼ 2.3	0.523	51.0 ± 7.9	−0.6	1.8	−4.2 ∼ 3.0	0.736
	12 W	55.6 ± 8.9	53.7 ± 8.9	−1.1	1.9	−4.9 ∼ 2.7	0.556	53.4 ± 7.6	−1.7	1.6	−5.0 ∼ 1.5	0.293
Vitality	0 W	45.8 ± 8.6	43.3 ± 6.7	−2.5	1.9	−6.4 ∼ 1.4	0.197	44.2 ± 9.3	−1.6	2.2	−6.1 ∼ 2.8	0.470
	12 W	49.7 ± 8.4	48.4 ± 9.6	−0.1	2.1	−4.3 ∼ 4.2	0.970	47.3 ± 9.5	−1.8	2.1	−5.9 ∼ 2.4	0.397
Social functioning	0 W	44.6 ± 11.1	46.4 ± 8.3	1.8	2.5	−3.1 ∼ 6.8	0.467	44.9 ± 8.2	0.3	2.4	−4.6 ∼ 5.2	0.899
	12 W	49.0 ± 11.7	50.7 ± 8.1	1.6	2.6	−3.5 ∼ 6.7	0.529	48.9 ± 8.8	−0.1	2.6	−5.2 ∼ 5.0	0.966
Role emotional	0 W	43.2 ± 8.9	44.4 ± 7.1	1.1	2.0	−3.0 ∼ 5.2	0.585	45.4 ± 9.3	2.2	2.3	−2.3 ∼ 6.7	0.339
	12 W	48.3 ± 9.3	48.3 ± 7.9	−0.3	2.1	−4.5 ∼ 4.0	0.896	49.0 ± 6.9	−0.1	1.8	−3.8 ∼ 3.5	0.945
Mental health	0 W	46.2 ± 5.4	47.3 ± 7.3	1.1	1.6	−2.1 ∼ 4.3	0.491	46.7 ± 6.9	0.5	1.5	−2.5 ∼ 3.6	0.739
	12 W	50.6 ± 8.9	51.9 ± 9.1	0.8	2.2	−3.6 ∼ 5.1	0.731	50.0 ± 8.1	−0.7	2.1	−4.9 ∼ 3.5	0.736

Data are given as mean ± standard deviation (SD). Statistical analysis was performed using ANCOVA for between-group comparisons after 12 weeks. pPE, porcine placenta extract; MD, Mean difference between groups; SE, Standard error; CI, Confidential interval; 0 W, baseline; 12 W, 12 weeks.

#### 3.3.3 Changes in the ogri-shirakawa-azumi sleep inventory MA version (OSA-MA) among participants

Reflection upon waking was assessed using five psychological scales. All groups showed an increase in the sleepiness on rising, initiation maintenance of sleep, frequent dreaming, and refreshing scores (Table 5). In contrast, changes in sleep length scores were not observed in any of the groups, as shown in Table 5. No significant differences were found between the placebo group and the group that ingested placenta in any of these items at 12 W (Table 5).

**TABLE 5:** Changes in sleep quality, as assessed using the Ogri-Shirakawa-Azumi sleep inventory MA version (OSA-MA), among participants.

Variable		Placebo group	pPE group					ePE group				
		Mean ± SD	Mean ± SD	MD (vs. placebo group)	SE (vs. placebo group)	95% CI (vs. placebo group)	*p*-value (vs. placebo group)	Mean ± SD	MD (vs. placebo group)	SE (vs. placebo group)	95% CI (vs. placebo group)	*p*-value (vs. placebo group)
Sleepiness on rising	0 W	14.8 ± 4.2	16.5 ± 4.5	1.7	1.1	−0.5 ∼ 3.9	0.137	14.1 ± 6.1	−0.7	1.3	−3.3 ∼ 1.9	0.573
	12 W	18.1 ± 5.0	17.5 ± 4.5	−1.5	1.1	−3.6 ∼ 0.6	0.169	18.1 ± 4.6	0.4	1.0	−1.5 ∼ 2.4	0.654
Initiation and maintenance of sleep	0 W	14.0 ± 5.6	16.8 ± 5.6	2.9	1,4	0.0 ∼ 5.7	0.047	13.9 ± 5.2	−0.1	1.4	−2.8 ∼ 2.6	0.953
	12 W	18.3 ± 4.6	19.0 ± 4.6	−0.3	1.1	−2.5 ∼ 1.8	0.750	17.0 ± 4.1	−1.3	0.9	−3.1 ∼ 0.6	0.188
Frequent dreaming	0 W	18.5 ± 8.5	19.8 ± 7.0	1.2	2.0	−2.7 ∼ 5.2	0.533	19.3 ± 6.5	0.8	1.9	−3.0 ∼ 4.6	0.683
	12 W	21.8 ± 7.2	22.8 ± 6.4	0.2	1.2	−2.2 ∼ 2.5	0.888	21.1 ± 6.3	−1.2	1.1	−3.4 ∼ 0.9	0.261
Refreshing	0 W	14.1 ± 4.8	14.1 ± 4.8	0.1	1.2	−2.3 ∼ 2.5	0.956	13.4 ± 5.9	−0.6	1.3	−3.3 ∼ 2.0	0.635
	12 W	16.8 ± 5.0	16.1 ± 4.9	−0.8	1.1	−3.0 ∼ 1.4	0.466	17.7 ± 4.6	1.2	1.0	−0.7 ∼ 3.2	0.218
Sleep length	0 W	17.2 ± 4.6	17.9 ± 5.3	0.7	1.3	−1.8 ∼ 3.2	0.572	17.0 ± 6.7	−0.1	1.4	−3.0 ∼ 2.7	0.925
	12 W	18.3 ± 4.6	19.0 ± 4.9	0.3	1.1	−1.8 ∼ 2.5	0.759	18.7 ± 4.6	0.4	1.0	−1.6 ∼ 2.5	0.667

Data are given as mean ± standard deviation (SD). Statistical analysis was performed using ANCOVA for between-group comparisons after 12 weeks. pPE, porcine placenta extract; MD, Mean difference between groups; SE, Standard error; CI, Confidential interval. 0 W: baseline. 12 W: 12 weeks.

#### 3.3.4 Changes in the autonomic nervous activity among participants

After 12 W, high frequency values decreased in the placebo and ePE groups, whereas they increased in the pPE group (Supplementary Table S3). The low frequency/high frequency ratio decreased in the placebo group, increased in both the pPE and ePE groups (Supplementary Table S3). Heart rate values significantly increased in the placebo group while decreasing in both the pPE group and the ePE group (Supplementary Table S3). The ccvTP was decreased in the placebo group. In contrast, there was no change in the pPE group and a decrease in the ePE group. No significant differences were found between the placebo group and the group that ingested placenta in any of these items at 12 W. (Supplementary Table S3).

#### 3.3 5 Changes in the plasma cortisol and DHEA-S among participants

After 12 W, DHEA-S levels decreased in all groups (Supplementary Table S4). Cortisol levels increased in the placebo group and decreased in both the pPE and ePE groups (Supplementary Table S4). No significant differences were found between the placebo and placenta ingestion groups at 12 W (Supplementary Table S4).

#### 3.3.6 Changes in the blood sex hormones among participants

At week 12, female estradiol and progesterone levels decreased in the placebo group, while they increased in the pPE and ePE groups (Supplementary Table S5); Follicle-stimulating hormone and luteinizing hormone levels increased in all groups, with luteinizing hormone increasing significantly in the ePE group (Supplementary Table S5). There was no significant difference between the placebo and pPE or ePE groups at week 12 (Supplementary Table S5). In males, free testosterone levels increased in all groups, with no significant differences observed within or between groups at 12 W (Supplementary Table S6).

#### 3.3.7 Safety assessment among participants

Blood biochemical parameters, including ALT, total protein, blood urea nitrogen, sodium, amylase, total cholesterol, triglycerides, and glucose, remained within reference ranges after 12 W of treatment (Supplementary Table S7). In addition, blood cell tests, such as white blood cell count, red blood cell count, hemoglobin content, hematocrit, and platelet count were all within reference limits (Supplementary Table S7). Importantly, no presumed study-related adverse events were observed in any of the groups during the study period.

## 4 Discussion

This study aimed to evaluate the effects of 12 W of pPE and ePE supplementation on mood, fatigue, quality of life, and sleep using subjective measures in healthy adults aged 20–65 years. pPE supplementation resulted in significant improvements in TMD and its subscales, including Anger-Hostility, Confusion-Bewilderment, Vigor-Activity, and Friendliness, compared to the placebo group. In contrast, supplementation with ePE showed non-significant improvements in these mood parameters. pPE or ePE supplementation was associated with non-significant improvements in fatigue, quality of life, and sleep compared with placebo.

In this study, post-intervention TMD scores were observed to be lower in the pPE and ePE groups compared to the placebo group, with a significant difference noted in the pPE group. TMD scores, derived from a standardized sample normalized to a mean and SD of 50 and 10, respectively, interpret lower scores as indicative of improved overall mood (33). Participants were selected based on relatively high baseline TMD scores, with mean scores above 50 for all groups, suggesting that the study population experienced higher-than-average negative mood states due to daily stress. In contrast, post-intervention scores for the placebo group remained above the standardized sample mean, while scores for the pPE and ePE groups declined below the standard sample mean, indicating clinically significant improvements in mood. Compared to the placebo group, the pPE group had significantly lower post-intervention scores for Anger-Hostility and Confusion-Bewilderment, while the ePE group showed no significant reductions. Anger-Hostility and Confusion-Bewilderment are psychological stress reactions commonly associated with emotions such as anger, hostility, and confusion (33, 34). The pPE group also had significantly higher post-intervention Vigor-Activity and Friendliness scores than the placebo group. While previous studies have reported significant increases in either Vigor-Activity or Friendliness scores with other interventions, significant improvements in both measures are rare (13, 35–37). Therefore, these results may be important as Vigor-Activity and Friendliness are strongly correlated with positive affect and inversely with negative affect (Anger-Hostility, Confusion-Bewilderment, Depression-Dejection, Fatigue-Inertia, and Tension-Anxiety) (33). TMD scores are calculated by subtracting the sum of the negative mood scale (Anger-Hostility, Confusion-Bewilderment, Depression-Dejection, Fatigue-Inertia, and Tension-Anxiety) scores from the positive mood scales (Vigor-Activity and Friendliness). In other words, pPE use may have improved overall mood by improving the stress response and suppressing negative emotions such as anger, hostility, confusion, and embarrassment. Subgroup analysis of POMS2 revealed similar trends in both men and women within the pPE intake group, suggesting that the overall effect of POMS2 can be generalized to both sexes.

Stress triggers physiological responses via the hypothalamic-pituitary-adrenal (HPA) axis, leading to the release of cortisol in humans (38). Cortisol has long been used as a biomarker of stress (39) and is inversely correlated with brain-derived neurotrophic factor (BDNF) in the nervous system (40). In the present study, 12 W of PE supplementation was found to decrease cortisol levels compared to the placebo group. In other words, it is speculated that pPE has a cortisol-regulating function. In addition, the pPE used in this study contains qualitative and quantitative BDNF-inducing dipeptides (Gly-Leu and Leu-Gly). It has been indicated that Gly-Leu and Leu-Gly activate neurons through promoting BDNF production (20). Although BDNF was not measured in this study, the improvement in TMD scores with pPE consumption may be related to cortisol regulation and increased BDNF production. Anger-Hostility, a component of the POMS scale, is a psychological phenotype associated with sympathetic activation. Stress-induced physiological changes are known to increase cortisol and adrenaline secretion (41). Since adrenaline promotes sympathetic activation, pPE supplementation may affect the autonomic nervous system as well as the HPA axis. The present study did not examine the effects of pPE on adrenaline levels, but the likelihood of pPE affecting the autonomic nervous system would be low, as noted above. However, further studies are needed to confirm these speculations, including measuring levels of BDNF and adrenaline.

A mixture of five amino acids, serine, alanine, glutamic acid, aspartic acid, and Tyr, has been reported to significantly improve motivation in the recovery period following a 4-week stressful mental work intervention (12). These findings suggest that amino acids may play a role as active compounds that contribute to mental health. However, the specific active components responsible for the positive effects of pPE and ePE supplementation on mood states, as observed in this study, have not yet been identified. Notably, these PEs are known to contain amino acids (42). Specifically, the PE used in our study contained the same amino acids as the aforementioned five-amino acid blend, and its intake was approximately three to eight times greater. Furthermore, in the previous study, significant differences in POMS2 scores were found in the before-and-after comparison, but not in the between-group comparison with the placebo group (12). On the other hand, in the present study, significant differences were observed in all POMS2 items in the before-and-after comparison and in five out of eight items in the between-group comparison with the placebo group. It is important to note the substantial difference in intervention periods between the two studies: 4 W in the former study and 12 W in the present study. Despite this disparity, the findings suggest that one of the active ingredients of the PE we used in the present study may be amino acids. Furthermore, while pig and horse PEs contain amino acids, they do not include tryptophan, a precursor for serotonin synthesis. On the other hand, Tyr is present in these PEs at about 5–8%, which raises the possibility that Tyr acts as a precursor to promote dopamine synthesis—an area that warrants further investigation.

BDNF is integral to neural regions and circuits (43) and plays an important role in mental stress (44). In addition, since BDNF can cross the blood-brain barrier (45), BDNF levels in the blood are reflective of brain BDNF levels (46, 47). Mental stress decreases BDNF synthesis in the brain (48), and work-related mental stress is associated with decreased blood BDNF in healthy individuals (49, 50). In addition, reduced BDNF is associated with depression (51), making BDNF a promising target for therapeutic intervention in mental stress-related disorders. Recently, it was reported that a 4-week intake of pPE containing Gly-Leu and Leu-Gly in healthy adult women significantly improved all POMS2 items compared to the placebo group, though no significant before-and-after differences were observed (21). This finding also suggests that an ingested component of pPE enhances BDNF production in human intestinal epithelial cells, promoting neuronal activation—a mechanism by which intestinal activity may regulate brain function. Specifically, the addition of pPE significantly promoted BDNF gene expression in human colon-derived cell lines (21). Moreover, oral administration of Gly-Leu or Leu-Gly in mice subjected to treadmill exercise-induced fatigue elevated dopamine, BDNF, and phosphorylated extracellular signal-regulated kinase levels, indicating these dipeptides contribute to BDNF production and potentially to mental health improvements (20). In a previous similar study, the daily intake of these dipeptides via PE was at least 0.46 mg (52). In the present study, the total intake of these dipeptides was at least 0.49 mg per day, nearly identical to that in the previous study. In both the previous and current studies, a comparison between 0 W and 12 W showed significant improvement in all eight POMS2 items (21). Moreover, while the previous study was conducted over 4 weeks and showed no significant improvement compared to the placebo group, the present study demonstrated significant improvement after 12 W of intake, suggesting that longer-term intake may have contributed to these differences, even considering variations between the two studies. It is further speculated that one of the mechanisms underlying the positive effects of the pPE used in this clinical trial on mood state is the activation of neurons via the enhancement of BDNF production by Gly-Leu and Leu-Gly. However, further studies are needed to support these speculations.

The pPE and ePE used in this study were extracts of placental tissues from different species, each processed using the same enzymatic digestion method. Therefore, both placental extracts likely contain a variety of components, including peptides, amino acids, nucleobases, and others (42, 53). However, given species differences, it can be inferred that not all components or active ingredients in pPE and ePE are necessarily identical, either qualitatively or quantitatively. Regarding their biological activities, pPE have been reported to improve menopausal symptoms (17) and skin quality in healthy women (54). On the other hand, ePE has been reported to inhibit UV-induced pigmentation in healthy women (55). However, drawing direct comparisons between the biological activities of pPE and ePE is challenging, as no studies have directly compared them within the same experimental framework. Nonetheless, this study offers some scientific insight into this area. The results suggest that pPE was more effective than ePE in improving POMS2 scores, supporting the speculation that both extracts may share common active ingredients but differ in their relative content. However, further studies are needed to confirm these speculations.

Environmental conditions such as temperature, humidity, and hours of sunlight have been reported to affect people’s mental health (56). The seasons in which this study was conducted were early summer and the rainy season in Tokyo. As all participants in this study resided in or around Tokyo and each clinical examination was conducted within a narrow timeframe of no more than 20 days, the study was carried out under nearly identical environmental conditions. Thus, seasonal effects can be excluded from the interpretation of the data.

## 5 Limitations and strength

A major limitation of this study was that the primary endpoints were based on subjective questionnaires. Plasma cortisol and DHEA-S, as biometric measures of stress, along with autonomic activity balance and ccvTP—objectively assessed using autonomic measurement sensors—could not compensate for the primary assessment results. The complete mechanism by which pPE supplementation improves mood remains unclear. Future studies incorporating BDNF measurements and analyses of changes in brain activity are needed to elucidate the mechanism of action underlying the anti-stress effects of pPE on the central nervous system. Other limitation of our study is the absence of *a priori* sample size calculation, which was not feasible due to budgetary constraints. However, a *post hoc* power analysis was performed to assess the statistical power based on the obtained data. However, the results showed a power of 53%. This is not an optimal result, and it is another limitation of the study. Furthermore, all of the participants in this study were Japanese. This could be a limitation when considering other geographical regions and cultures.

## 6 Conclusion

The results of this study highlight the potential of pPE (1,000 mg) in alleviating negative emotions (such as anger, hostility, confusion, and embarrassment) while promoting positive emotions (such as liveliness, vitality, and friendliness) in response to daily stress. These findings suggest that pPE consumption may help maintain good mental health and positively impact lifestyle improvement. Future studies should adopt a crossover design to better define the optimal dosage and efficacy of pPE. Further research is also needed to elucidate the mechanism of action of pPE and to identify the active components (such as amino acids and peptides) responsible for its effects.

## Data Availability

The raw data supporting the conclusions of this article will be made available by the authors, without undue reservation.
