# Supplementary material for: Supplementation with porcine placenta extract reduces negative emotions and enhances positive emotions in healthy adults: a randomized, double-blind, placebo-controlled study
Source: Front Nutr. 2025 Jul 23;12:1570736. doi: 10.3389/fnut.2025.1570736 (PMC12325048; doi:10.3389/fnut.2025.1570736)
Supplement: Supplementary file 1 [file Table_1.docx]

Supplementation with porcine placenta extract reduces negative emotions and enhances positive emotions in healthy adults: A randomized, double-blind, placebo-controlled study

Supplementary Material

# 1 Supplementary Data

**Table S1. Subgroup analysis of change in participants' POMS2 scores.**

| **Variable** |  | **Placebo**  **(Mean ± SD)** | **pPE**  **(Mean ± SD)** | **MD**  **(vs. placebo group)** | **SE**  **(vs. placebo group)** | **95% CI**  **(vs. placebo group)** | ***p* Value**  **(vs. placebo group)** |
| --- | --- | --- | --- | --- | --- | --- | --- |
| TMD (female) | 0W | 52.6 ± 7.6 | 51.5 ± 5.9 | -1.1 | 2.1 | -5.3 ~ 3.1 | 0.599 |
|  | 12W | 51.2 ± 11.2 | 46.8 ± 7.5 | -3.1 | 1.8 | -6.7 ~ 0.4 | 0.083 |
| TMD (male) | 0W | 56.0 ± 8.3 | 54.6 ± 7.2 | -1.4 | 3.6 | -9.0 ~ 6.1 | 0.691 |
|  | 12W | 52.2 ± 8.7 | 48.7 ± 7.6 | -2.2 | 1.9 | -6.3 ~ 1.9 | 0.271 |
| Anger-Hostility (female) | 0W | 51.0 ± 9.8 | 48.0 ± 6.7 | -3.1 | 2.6 | -8.3 ~ 2.1 | 0.235 |
|  | 12W | 50.3 ± 11.0 | 44.8 ± 5.4 | -3.4 | 2.0 | -7.5 ~ 0.6 | 0.096 |
| Anger-Hostility (male) | 0W | 50.5 ± 11.3 | 51.4 ± 7.4 | 0.9 | 4.3 | -8.3 ~ 10.2 | 0.831 |
|  | 12W | 50.4 ± 11.4 | 47.4 ± 8.0 | -3.5 | 3.9 | -11.8 ~ 4.9 | 0.389 |
| Confusion-Bewilderment (female) | 0W | 53.0 ± 8.4 | 53.1 ± 8.0 | 0.1 | 2.5 | -4.9 ~ 5.1 | 0.958 |
|  | 12W | 54.0 ± 12.5 | 48.0 ± 8.5 | -6.1 | 2.2 | -10.5 ~ 1.7 | 0.008** |
| Confusion-Bewilderment (male) | 0W | 58.3 ± 8.7 | 54.7 ± 6.8 | -3.6 | 3.6 | -11.2 ~ 3.9 | 0.325 |
|  | 12W | 53.7 ± 9.0 | 49.6 ± 8.5 | -0.7 | 2.2 | -5.4 ~ 4.0 | 0.760 |
| Vigor-Activity (female) | 0W | 47.6 ± 9.5 | 46.4 ± 7.9 | -1.1 | 2.6 | -6.5 ~ 4.2 | 0.670 |
|  | 12W | 51.4 ± 10.7 | 53.6 ± 9.9 | 3.0 | 2.6 | -2.2 ~ 8.2 | 0.253 |
| Vigor-Activity (male) | 0W | 46.6 ± 9.8 | 43.9 ± 7.7 | -2.7 | 4.0 | -11.2 ~ 5.8 | 0.511 |
|  | 12W | 47.9 ± 7.8 | 53.8 ± 10.8 | 8.2 | 2.6 | 2.7 ~ 13.7 | 0.006** |
| Friendliness (female) | 0W | 51.4 ± 11.4 | 50.9 ± 10.6 | -0.5 | 3.3 | -7.2 ~ 6.2 | 0.889 |
|  | 12W | 50.7 ± 10.9 | 54.9 ± 9.4 | 4.6 | 2.2 | 0.2 ~ 8.9 | 0.041* |
| Friendliness (male) | 0W | 51.4 ± 8.4 | 48.1 ± 6.5 | -3.3 | 3.4 | -10.6 ~ 4.0 | 0.353 |
|  | 12W | 52.5 ± 8.3 | 54.0 ± 6.1 | 3.0 | 3.1 | -3.7 ~ 9.6 | 0.359 |

Data are given as mean ± standard deviation (SD). Statistical analysis was performed using ANCOVA for between-group comparisons after 12 weeks. pPE: porcine placenta extract. MD: Mean difference between groups. SE; Standard error. CI: Confidential interval. 0W: baseline. 12W: 12 weeks. TMD: Total Mood Disturbance. *: *p* < 0.05 vs. placebo group. **: *p* < 0.01 vs. placebo group.

**Table S2. Proportion of responders at 12 W in the placebo and pPE groups in POMS2 scores.**

| **Variable** |  | **Beneficial changes** | **No meaningful changes** | **Adverse changes** |
| --- | --- | --- | --- | --- |
| TMD | Placebo | 14 (45%) | 12 (39%) | 5 (16%) |
|  | pPE | 22 (69%) | 8 (25%) | 2 (6%) |
| Anger-Hostility | Placebo | 5 (16%) | 22 (71%) | 4 (13%) |
|  | pPE | 7 (22%) | 23(72%) | 2 (6%) |
| Confusion-Bewilderment | Placebo | 8 (26%) | 18 (58%) | 5 (16%) |
|  | pPE | 18 (56%) | 11 (34%) | 3(9%) |
| Vigor-Activity | Placebo | 12 (39%) | 14(45%) | 5 (16%) |
|  | pPE | 18 (56%) | 13 (41%) | 1 (3%) |
| Friendliness | Placebo | 5 (16%) | 19 (62%) | 7(23%) |
|  | pPE | 14 (44%) | 16 (50%) | 2 (6%) |

Data are given as n (%). pPE: porcine placenta extract. TMD: Total Mood Disturbance.

**Table S3.** Autonomic nervous activity evaluation of the placebo, pPE, and ePE groups at 0W and 12W.

|  |  | **Placebo**  **(Mean ± SD)** | **pPE**  **(Mean ± SD)** | **MD**  **(vs. placebo group)** | **SE**  **(vs. placebo group)** | **95% CI**  **(vs. placebo group)** | ***p* Value**  **(vs. placebo group)** | **ePE**  **(Mean ± SD)** | **MD**  **(vs. placebo group)** | **SE**  **(vs. placebo group)** | **95% CI**  **(vs. placebo group)** | ***p* Value**  **(vs. placebo group)** |
| --- | --- | --- | --- | --- | --- | --- | --- | --- | --- | --- | --- | --- |
| High frequency (ms^2^) | 0W | 208.9 ± 174.6 | 282.1 ± 313.0 | 73.1 | 63.6 | -54.7 ~ 201.0 | 0.256 | 306.0 ± 388.2 | 97.1 | 73.6 | -51.0 ~ 245.2 | 0.194 |
|  | 12W | 156.4 ± 170.1 | 372.2 ± 639.6 | 162.2 | 111.1 | -60.1 ~ 384.5 | 0.150 | 225.4 ± 292.3 | 52.4 | 59.9 | -67.3 ~ 172.1 | 0.385 |
| Ratio of low frequency / high frequency | 0W | 4.5 ± 8.9 | 2.8 ± 4.3 | -1.8 | 1.8 | -5.3 ~ 1.8 | 0.326 | 2.1 ± 2.4 | -2.4 | 1.7 | -5.7 ~ 1.0 | 0.162 |
|  | 12W | 2.9 ± 3.6 | 3.3 ± 4.5 | 0.8 | 0.9 | -1.1 ~ 2.7 | 0.391 | 3.0 ± 3.4 |  |  | -54.7 ~ 201.0 | 0.475 |
| Heart rate (bpm) | 0W | 73.1 ± 10.0 | 76.9 ± 13.6 | 3.8 | 3.0 | -2.2 ~ 9.8 | 0.205 | 78.1 ± 13.7 |  |  | -54.7 ~ 201.0 | 0.093 |
|  | 12W | 77.6 ± 9.7 | 75.8 ± 11.4 | -3.9 | 2.2 | -8.2 ~ 0.4 | 0.076 | 76.5 ± 9.5 |  |  | -54.7 ~ 201.0 | 0.034* |
| ccvTP | 0W | 2.5 ± 1.0 | 2.8 ± 1.8 | 0.3 | 0.4 | -0.4 ~ 1.0 | 0.418 | 2.9 ± 1.6 |  |  | -54.7 ~ 201.0 | 0.281 |
|  | 12W | 2.2 ± 1.1* | 2.8 ± 1.8 | 0.5 | 0.3 | -0.1 ~ 1.1 | 0.126 | 2.5 ± 1.2 |  |  | -54.7 ~ 201.0 | 0.417 |

Data are given as mean ± standard deviation (SD). Statistical analysis was performed using ANCOVA for between-group comparisons after 12 weeks. pPE: porcine placenta extract. ePE: equine placenta extract. MD: Mean difference between groups. SE; Standard error. CI: Confidential interval. 0W: baseline. 12W: 12 weeks. ccvTP: coefficient of component variance for Total Power. *: *p* < 0.05 vs. placebo group.

**Table S4.** Changes in the plasma cortisol, DHEA-S, and cortisol/DHEA-S ratio between the placebo, pPE, and ePE groups in females at 0W and 12W.

| **Variable** |  | **Placebo**  **(Mean ± SD)** | **pPE**  **(Mean ± SD)** | **MD**  **(vs. placebo group)** | **SE**  **(vs. placebo group)** | **95% CI**  **(vs. placebo group)** | ***p* Value**  **(vs. placebo group)** | **ePE**  **(Mean ± SD)** | **MD**  **(vs. placebo group)** | **SE**  **(vs. placebo group)** | **95% CI**  **(vs. placebo group)** | ***p* Value**  **(vs. placebo group)** |
| --- | --- | --- | --- | --- | --- | --- | --- | --- | --- | --- | --- | --- |
| DHEA-S (μg/dL) | 0W | 186.1 ± 144.6 | 193.9 ± 96.5 | 0.9 | 0.8 | -0.7 ~ 2.6 | 0.801 | 170.7 ± 96.6 | -15.3 | 30.8 | -77.2 ~ 46.5 | 0.621 |
|  | 12W | 170.6 ± 127.4 | 180.3 ± 105.4 | -0.3 | 0.7 | -1.7 ~ 1.1 | 0.802 | 153.3 ± 98.0 | -4.0 | 10.0 | -24.0 ~ 15.9 | 0.686 |
| Cortisol (μg/dL) | 0W | 9.0 ± 2.4 | 9.9 ± 4.0 | 7.9 | 31.1 | -54.5 ~ 70.2 | 0.263 | 9.2 ± 3.3 | 0.2 | 0.7 | -1.2 ~ 1.6 | 0.744 |
|  | 12W | 9.3 ± 3.3 | 9.4 ± 2.9 | 2,7 | 10.7 | -18.7 ~ 24.2 | 0.666 | 8.5 ± 3.2 | -0.8 | 0.8 | -2.3 ~ 0.7 | 0.290 |

Data are given as mean ± standard deviation (SD). Statistical analysis was performed using ANCOVA for between-group comparisons after 12 weeks. pPE: porcine placenta extract. ePE: equine placenta extract. MD: Mean difference between groups. SE; Standard error. CI: Confidential interval. 0W: baseline. 12W: 12 weeks. DHEA-S: dehydroepiandrosterone sulphate.

**Table S5.** Changes in oestradiol, FSH, luteinizing hormone, and progesterone between the placebo, pPE, and ePE groups in females at 0W and 12W.

| **Variable** |  | **Placebo**  **(n = 21)** | **pPE**  **(n = 23)** | **MD**  **(vs. placebo group)** | **SE**  **(vs. placebo group)** | **95% CI**  **(vs. placebo group)** | ***p* Value**  **(vs. placebo group)** | **ePE**  **(n = 24)** | **MD**  **(vs. placebo group)** | **SE**  **(vs. placebo group)** | **95% CI**  **(vs. placebo group)** | ***p* Value**  **(vs. placebo group)** |
| --- | --- | --- | --- | --- | --- | --- | --- | --- | --- | --- | --- | --- |
| Oestradiol (pg/mL) | 0W | 64.2 ± 122.1 | 63.0 ± 6.1 | -1.3 | 32.1 | -66.5 ~ 63.9 | 0.968 | 89.5 ± 106.9 | 25.2 | 34.4 | -44.4 ~ 94.8 | 0.468 |
|  | 12W | 36.1 ± 75.9 | 169.6 ± 454.6 | 133.7 | 101.6 | -71.5 ~ 339.0 | 0.195 | 111.1 ± 165.0 | 63.0 | 36.4 | -10.4 ~ 136.3 | 0.091 |
| Follicle-stimulating hormone (mIU/mL) | 0W | 43.8 ± 37.0 | 23.6 ± 28.7 | -20.3 | 10.0 | -40.6 ~ 0.0 | 0.050 | 27.1 ± 32.9 | -16.7 | 10.5 | -37.9 ~ 4.5 | 0.118 |
|  | 12W | 48.6 ± 37.3 | 25.7 ± 29.6 | -4.1 | 4.4 | -13.1 ~ 4.8 | 0.359 | 27.3 ± 29.9 | -6.3 | 3.9 | -14.2 ~ 1.5 | 0.110 |
| Luteinizing hormone (mIU/mL) | 0W | 16.9 ± 11.8 | 8.9 ± 9.7 | -8.0 | 3.3 | -14.6 ~ -1.4 | 0.019 | 10.7 ± 11.1 | -6.2 | 3.4 | -13.1 ~ 0.7 | 0.079 |
|  | 12W | 17.9 ± 12.5 | 9.6 ± 9.9 | -1.3 | 2.0 | -5.3 ~ 2.8 | 0.528 | 13.6 ± 11.1 | 0.8 | 2.2 | -3.5 ~ 5.2 | 0.705 |
| Progesterone (ng/mL) | 0W | 0.9 ± 3.0 | 2.0 ± 4.0 | 1.1 | 1.1 | -1.0 ~ 3.3 | 0.293 | 3.3 ± 6.5 | 2.4 | 1.5 | -0.6 ~ 5.4 | 0.117 |
|  | 12W | 0.2 ± 0.5 | 3.0 ± 6.7 | 2.6 | 1.5 | -0.4 ~ 5.6 | 0.088 | 1.9 ± 5.1 | 1.8 | 1.2 | -0.5 ~ 4.2 | 0.117 |

Data are given as mean ± standard deviation (SD). Statistical analysis was performed using ANCOVA for between-group comparisons after 12 weeks. pPE: porcine placenta extract. ePE: equine placenta extract. MD: Mean difference between groups. SE; Standard error. CI: Confidential interval. 0W: baseline. 12W: 12 weeks.

**Table S6.** Changes in free testosterone between the placebo, pPE, and ePE groups in males at 0W and 12W.

| **Variable** |  | **Placebo**  **(n = 10)** | **pPE**  **(n = 9)** | **MD**  **(vs. placebo group)** | **SE**  **(vs. placebo group)** | **95% CI**  **(vs. placebo group)** | ***p* Value**  **(vs. placebo group)** | **ePE**  **(n = 10)** | **MD**  **(vs. placebo group)** | **SE**  **(vs. placebo group)** | **95% CI**  **(vs. placebo group)** | ***p* Value**  **(vs. placebo group)** |
| --- | --- | --- | --- | --- | --- | --- | --- | --- | --- | --- | --- | --- |
| Free testosterone (pg/mL) | 0W | 8.5 ± 4.9 | 11.7 ± 4.1 | 3.2 | 2.1 | -1.1 ~ 7.6 | 0.137 | 10.5 ± 2.7 | 2.0 | 1.8 | -1.8 ~ 5.8 | 0.275 |
|  | 12W | 9.9 ± 5.2 | 12.7 ± 2.6 | 1.1 | 1.7 | -2.6 ~4.8 | 0.523 | 11.1 ± 4.8 | -0.4 | 1.8 | -4.2 ~ 3.5 | 0.844 |

Data are given as mean ± standard deviation (SD). Statistical analysis was performed using ANCOVA for between-group comparisons after 12 weeks. pPE: porcine placenta extract. ePE: equine placenta extract. MD: Mean difference between groups. SE; Standard error. CI: Confidential interval. 0W: baseline. 12W: 12 weeks.

**Table S7.** Safety evaluation of the placebo, pPE, and ePE groups at 0W and 12W.

| Variable |  | Reference  value | Placebo  (Mean ± SD) | pPE  (Mean ± SD) | ePE  (Mean ± SD) |
| --- | --- | --- | --- | --- | --- |
| WBC (/μL) | 0W | 3300.0 - 8600.0 | 5232.0 ± 1345.0 | 5334.4 ± 1516.0 | 6079.0 ± 1917.0 |
|  | 12W |  | 5048.0 ± 1210.0 | 5371.9 ± 1440.0 | 5697.0 ± 1445.0 |
| RBC (×10^4^/μL) | 0W | 386.0 - 555.0 | 442.0 ± 52.0 | 438.1 ± 47.0 | 459.0 ± 48.0 |
|  | 12W |  | 437.0 ± 47.0 | 431.8 ± 44.0 | 443.0 ± 45.0 |
| Hb (g/dL) | 0W | 11.6 - 16.8 | 13.4 ± 1.4 | 13.1 ± 1.6 | 14.0 ± 1.5 |
|  | 12W |  | 13.2 ± 1.4 | 13.0 ± 1.5 | 13.4 ± 1.4 |
| Ht (%) | 0W | 35.1 – 50.1 | 41.4 ± 4.2 | 40.9 ± 4.6 | 43.0 ± 4.2 |
|  | 12W |  | 41.7 ± 4.0 | 41.4 ± 4.4 | 42.5 ± 4.1 |
| PLT (×10^4^/μL) | 0W | 15.8 – 34.8 | 27.1 ± 7.2 | 27.7 ± 5.2 | 28.6 ± 8.1 |
|  | 12W |  | 27.1 ± 6.8 | 27.3 ± 5.1 | 26.4 ± 6.7 |
| AST (U/L) | 0W | 13.0 – 33.0 | 22.0 ± 7.0 | 20.2 ± 8.0 | 22.0 ± 6.0 |
|  | 12W |  | 24.0 ± 13.0 | 20.3 ± 10.0 | 23.0 ± 14.0 |
| ALT (U/L) | 0W | 6.0 – 42.0 | 22.0 ± 17.0 | 16.1 ± 6.0 | 20.0 ± 10.0 |
|  | 12W |  | 22.0 ± 28.0 | 15.4 ± 5.0 | 20.0 ± 15.0 |
| GGT (U/L) | 0W | 10.0 – 47.0 | 28.0 ± 28.0 | 27.8 ± 44.0 | 29.0 ± 25.0 |
|  | 12W |  | 26.0 ± 23.0 | 31.9 ± 61.0 | 35.0 ± 40.0 |
| T-BIL (mg/dL) | 0W | 0.3 – 1.2 | 0.8 ± 0.3 | 0.7 ± 0.3 | 0.8 ± 0.2 |
|  | 12W |  | 0.8 ± 0.3 | 0.8 ± 0.2 | 0.8 ± 0.3 |
| TP (g/dL) | 0W | 6.7 – 8.3 | 7.0 ± 0.5 | 7.2 ± 0.4 | 7.3 ± 0.4 |
|  | 12W |  | 7.1 ± 0.5 | 7.2 ± 0.3 | 7.1 ± 0.3 |
| BUN (mg/dL) | 0W | 8.0 – 22.0 | 12.8 ± 3.8 | 12.6 ± 2.9 | 11.9 ± 2.5 |
|  | 12W |  | 13.5 ± 3.8 | 12.3 ± 3.9 | 12.4 ± 3.0 |
| CRE (mg/dL) | 0W | 0.4 – 1.1 | 0.7 ± 0.2 | 0.7 ± 0.2 | 0.7 ± 0.1 |
|  | 12W |  | 0.7 ± 0.2 | 0.7 ± 0.2 | 0.7 ± 0.1 |
| UA (mg/dL) | 0W | 2.3 – 7.0 | 4.9 ± 1.4 | 4.7 ± 1.4 | 4.4 ± 1.2 |
|  | 12W |  | 5.1 ± 1.4 | 4.7 ± 1.3 | 4.5 ± 1.2 |
| Na (mEq/L) | 0W | 138.0 – 146.0 | 142.0 ± 2.0 | 140.4 ± 2.0 | 141.0 ± 2.0 |
|  | 12W |  | 141.0 ± 2.0 | 140.9 ± 2.0 | 141.0 ± 2.0 |
| K (mEq/L) | 0W | 3.6 – 4.9 | 4.4 ± 0.4 | 4.4 ± 0.4 | 4.5 ± 0.4 |
|  | 12W |  | 4.1 ± 0.4 | 4.1 ± 0.4 | 4.1 ± 0.4 |
| Cl (mEq/L) | 0W | 99.0 – 109.0 | 103.0 ± 2.0 | 102.4 ± 3.0 | 102.0 ± 2.0 |
|  | 12W |  | 103.0 ± 2.0 | 102.3 ± 2.0 | 102.0 ± 2.0 |
| AMY/S (U/L) | 0W | 37.0 – 125.0 | 81.0 ± 23.0 | 78.9 ± 32.0 | 82.0 ± 30.0 |
|  | 12W |  | 75.0 ± 17.0 | 74.6 ± 31.0 | 77.0 ± 25.0 |
| T-Cho (mg/dL) | 0W | 128.0 – 219.0 | 206.0 ± 34.0 | 204.5 ± 36.0 | 210.0 ± 53.0 |
|  | 12W |  | 205.0 ± 34.0 | 201.3 ± 39.0 | 199.0 ± 44.0 |
| HDL-Cho (mg/dL) | 0W | 40.0 – 96.0 | 73.0 ± 18.0 | 75.1 ± 22.0 | 74.0 ± 22.0 |
|  | 12W |  | 72.0 ± 17.0 | 75.4 ± 23.0 | 70.0 ± 18.0 |
| LDL-Cho (mg/dL) | 0W | 140 > | 116.0 ± 29.0 | 113.4 ± 31.0 | 119.0 ± 43.0 |
|  | 12W |  | 112.0 ± 29.0 | 109.4 ± 32.0 | 112.0 ± 37.0 |
| TG (mg/dL) | 0W | 30.0 – 149.0 | 87.0 ± 60.0 | 82.0 ± 44.0 | 83.0 ± 52.0 |
|  | 12W |  | 102.0 ± 100.0 | 73.0 ± 33.0 | 75.0 ± 36.0 |
| GLU (mg/dL) | 0W | 80.0 – 112.0 | 89.0 ± 12.0 | 88.8 ± 13.0 | 85.0 ± 8.0 |
|  | 12W |  | 89.0 ± 11.0 | 87.8 ± 12.0 | 88.0 ± 22.0 |
| HbA1c (%) | 0W | 4.6 – 6.2 | 5.3 ± 0.4 | 5.3 ± 0.2 | 5.3 ± 0.2 |
|  | 12W |  | 5.4 ± 0.4 | 5.4 ± 0.3 | 5.4 ± 1.0 |

Data are given as mean ± standard deviation (SD). pPE: porcine placenta extract. ePE: equine placenta extract. 0W: baseline. 12W: 12 weeks. WBC, White blood cells; RBC, Red blood cells; Hb, Haemoglobin; Ht, Haematocrit; PLT, Platelets; AST, Aspartate aminotransferase; ALT, Alanine aminotransferase; GGT, Gamma-glutamyl transferase; T-BIL, Total bilirubin; TP, Total protein; BUN, Blood urea nitrogen; CRE, Creatinine; UA, Uric acid; Na, Sodium; K, Potassium; Cl, Chloride; AMY/S, Salivary amylase; T-Cho, Total cholesterol; HDL-Cho, High density lipoprotein cholesterol; LDL-Cho, Low density lipoprotein cholesterol; TG, Triglycerides; GLU, Glucose; HbA1c, Haemoglobin A1c.
